# Investigating Dishonesty-Does Context Matter?

**DOI:** 10.3389/fpsyg.2021.684735

**Published:** 2021-08-23

**Authors:** Aline Waeber

**Affiliations:** Institute of Insurance Economics, University of St. Gallen, St. Gallen, Switzerland

**Keywords:** lying, honesty, moral behavior, framing, context-dependence

## Abstract

This paper introduces frame-specific randomization devices to vary the situational context of an online lying experiment. Participants are asked to report outcomes of random draws from two different sources of uncertainty—decimals of the value of a stock index or a neutrally framed random number generator. The findings show that the frame-specific randomization device is not prone to the social norm effects documented in the literature. Because different environments can evoke different norms, I replicate the experiment in the more constrained setting of a traditional physical laboratory revealing no systematic differences in behavior. Furthermore, I am not able to show that participants who take longer to report are more honest and this is specific to the physical laboratory environment. Finally, the findings reveal gender differences in honesty depending on the environment—males are more honest when they participate in the laboratory as opposed to online.

## 1. Introduction

This research mainly centers around two different research questions addressed in two experimental studies. In the first study, I estimate whether financial market saliency triggers dishonest behavior in an online experiment. More specifically, the experimental design allows to test whether participants are more honest when they are introduced to a financial market context as opposed to a neutral context. I use frame-specific randomization devices to vary the situational context of the game (i.e., stock market or neutral context). Although most of standard economic theory implicitly assumes that people act as if preferences are stable, there is abundant evidence (Tversky and Kahneman, 1981; Dufwenberg et al., 2011) showing that subtle differences in the way a situation is framed can cause changes in preferences^1^. To develop a deeper understanding of the causes of potential differences in behavior when the source of uncertainty is a stock market index, I elicit individual beliefs about dishonest behavior of others. In the second study, I aim to find out whether the environment (i.e., physical laboratory or online) has an effect on dishonest behavior. In both studies, the dependent variable is the reported draw defined on the interval between 0 and 9. I additionally capture the variation in behavior that is induced not only by the previously mentioned independent variables (i.e., financial market setting/environment), but also decision times.

Previous research suggests that different environments evoke different norms of behavior. The stock market environment may be linked to contexts in which competitive or exploitative norms prevail (Liberman et al., 2004; Cohn et al., 2014). This means that the stock market context may trigger a stronger desire to be greedy. Participants in the stock market context could thus feel as it is easier to justify dishonest behavior to increase payoffs if he/she believes the norm in that specific environment is to make as much money as possible. In contrast, the neutral setting should not evoke any strong connotations (Cohn et al., 2014). I replicate the experiment in the more constrained setting of the traditional physical laboratory. It is possible that participants feel more socially distant from others in an online environment^2^. This might reduce participants' need to adhere to social norms of behavior. Another source of variation in dishonest behavior (though endogenous) is the time it takes to make a decision. I thus explore differences in decision times depending on the environment (i.e., laboratory or online). A recent meta-analysis finds that honesty is deliberative (Köbis et al., 2019). I thus expect decision times and dishonesty to be negatively correlated.

The results from the online experiment show no significant differences in dishonest behavior between the two enviroments—stock market and neutral. This indicates that this specific source of uncertainty is not prone to the social norm effects documented in the literature. The findings confirm previous studies that do not find significant differences in a student sample between a financial and a neutral context (Cappelen et al., 2013; Huber and Huber, 2020). More specifically, the frame-specific device does not shift participants' beliefs about the prevailing honesty norm. Furthermore, there are no significant differences in dishonest behavior conditional on the environment (i.e., physical lab or online). Looking at decision times, I find that participants who take longer to report are more honest. However, this is only true for subjects in the physical laboratory—a possible sign of self-reflection of self-image violating behavior. Finally, the results suggest gender differences in honest behavior depending on the environment. Even the slightest cues of being observed seem to affect male but not female reporting behavior.

**Related Literature**. The study first and foremost relates to the literature on framing effects in social preference games. Framing generally refers to the observation that a decision problem can be presented in different ways, for example, in positive or negative connotations or “frames”^3^. Of particular importance are studies contrasting conditions in which the description of the relevant task evokes norms related to competitive vs. cooperative norms. Earlier work reveals that people cooperate more in a prisoner's dilemma when it is called the *Community Game* than when it is called the *Wall Street Game* (Kay and Ross, 2003; Liberman et al., 2004; Ellingsen et al., 2012). However, social framing effects in the prisoner's dilemma vanish when the game is played sequentially. This suggests that social cues primarily work by changing participants' beliefs about other people in the interaction rather than participants' preferences (Fehr and Schmidt, 2006; Ellingsen et al., 2012). Dreber et al. (2013) investigate whether social framing effects are also present in dictator games. They find that dictators are not sensitive to different frames. Contrary to this, Chang et al. (2019) do find an effect in a politically framed dictator game. They vary whether participants are shown neutrally framed or tax-framed dictator games. The aim is to render a U.S. political identity salient (i.e., Democrat or Republican) and to evoke the associated norm for that identity. They show that framing causes participants to apply different norms to the situation which affects their behavior. Andreoni (1995) finds significant differences in contributions when a public goods game is framed as *giving to a public good* as opposed to *taking from a public good*. In Krupka and Weber (2013), when the dictator game is framed as taking from another's endowment (i.e., a bully game) as opposed to giving away a portion of one's own endowment (i.e., a standard dictator game), bullies claimed less than did dictators. Thus, these findings indicate that changes in norms induce changes in behavior in otherwise identical economic games. Similarly, Capraro and Vanzo (2019) show that the words used to describe the available actions can affect people's decisions in extreme dictator games. However, in their study, the *take* frame does not give rise to a rate of pro-sociality significantly higher than the *give* frame.

Regarding the effect of financial market saliency on dishonest behavior, the evidence is mixed. Research from priming studies finds that simply priming subjects with the concept of money evokes more selfish behavior (Vohs et al., 2008; Vohs, 2015). In a subsequent study, Cohn et al. (2014) find that when financial professionals are reminded of their professional identity, they become more dishonest than their colleagues who are asked to think about leisure activities. The authors argue that “the prevailing business culture in the banking industry weakens and undermines the honesty norm.” However, more recent studies challenge these findings. For example, Rahwan et al. (2019) failed to replicate the results of more dishonest behavior among bankers across several populations. Rahwan et al. (2019) argue that differences in honesty could be attributed to heterogeneity in national banking norms, especially heterogeneity in the general population's relative expectation of bankers.^4^. Other studies point out that using a neutral prime for the control group (instead of *leisure activities*) might change results (Stöckl, 2015; Vranka and Houdek, 2015). Framing their experiment in a financial context, Huber and Huber (2020) show that financial professionals act more honestly in a financial context as opposed to a neutral context. However, this difference in behavior cannot be confirmed within a sample of students. The authors identify reputational concerns as one of the drivers of financial professionals' behavior. Similarly, Cappelen et al. (2013) find that students do not lie significantly less when they are in a market context. The above-mentioned studies vary the name attached to a game, while I vary the situational context of the game using a frame-specific device.

My work is furthermore related to the literature focusing on the psychological costs of dishonesty. The recent experimental literature has shown that individuals are often willing to forego financial benefits to behave honestly (Gneezy, 2005; Mazar et al., 2008; Erat and Gneezy, 2012; Cappelen et al., 2013; Fischbacher and Föllmi-Heusi, 2013; Abeler et al., 2014; Gneezy et al., 2018). The literature on intrinsic costs of lying suggests that people have internal standards for honesty which influence their self-concept (see Mazar et al., 2008). These internal standards are shaped by the norms and values of a society (Henrich et al., 2001). People thus do not only consider the expected monetary gains from lying, the probability of being caught, and the potential punishment but also how the act of lying might make them perceive themselves. This means that people do lie when it pays, but only to the extent that their perception of themselves as an honest person is not violated. Analyzing dishonesty in low stake scenarios, Barron (2019) shows that a substantial fraction of subjects lie downwards (i.e., giving up money to signal honesty). These subjects care about appearing good in more lucrative interactions^5^.

Fraud and unethical behavior are recurring issues in markets, which are costly for all market participants. Dishonesty poses a severe negative externality to markets, which can ultimately cause market failure. If everyone behaves honestly, eveyone benefits because high costs arise in doing business otherwise. An example of everyday deception is insurance fraud. The FBI estimates the total cost of insurance fraud in the U.S. (non-health insurance) to be more than USD 40 billion per year, which increases premiums for the average U.S. family between USD 400 and USD 700 annually (FBI, 2020). Similar acts of dishonesty can be observed in tax reporting. A recent Internal Revenue Service (IRS) study estimates the tax gap (i.e., the difference between what the IRS estimates taxpayers should pay and what they actually pay) at USD 441 billion per year for the 2011–2013 timeframe (Internal Revenue Service, 2019).

## 2. Methods-Experimental Design

I present a novel experimental design to measure dishonest behavior in an online setting. The experimental task is a one-shot individual decision-making situation. I rely on a between-subjects design in which the treatments are distinguished by how the particular decision situation is framed (i.e., random number, financial market).

The experiment follows the fundamental idea of other experimental setups to infer dishonest behavior (e.g., Fischbacher and Föllmi-Heusi, 2013) by asking participants to report a randomly generated number^6^. I collect reports of unobserved payout-determining random draws from two novel non-physical and verifiably random sources of uncertainty. I let participants report the outcomes of random draws from either decimals of a stock index price (*T*_*FM*_) or a random number generator (*T*_*RN*_). As mentioned earlier, participants took part in the study not in the laboratory but at home in the main part of this study.

### 2.1. Treatment Variations

**Figure 1:**
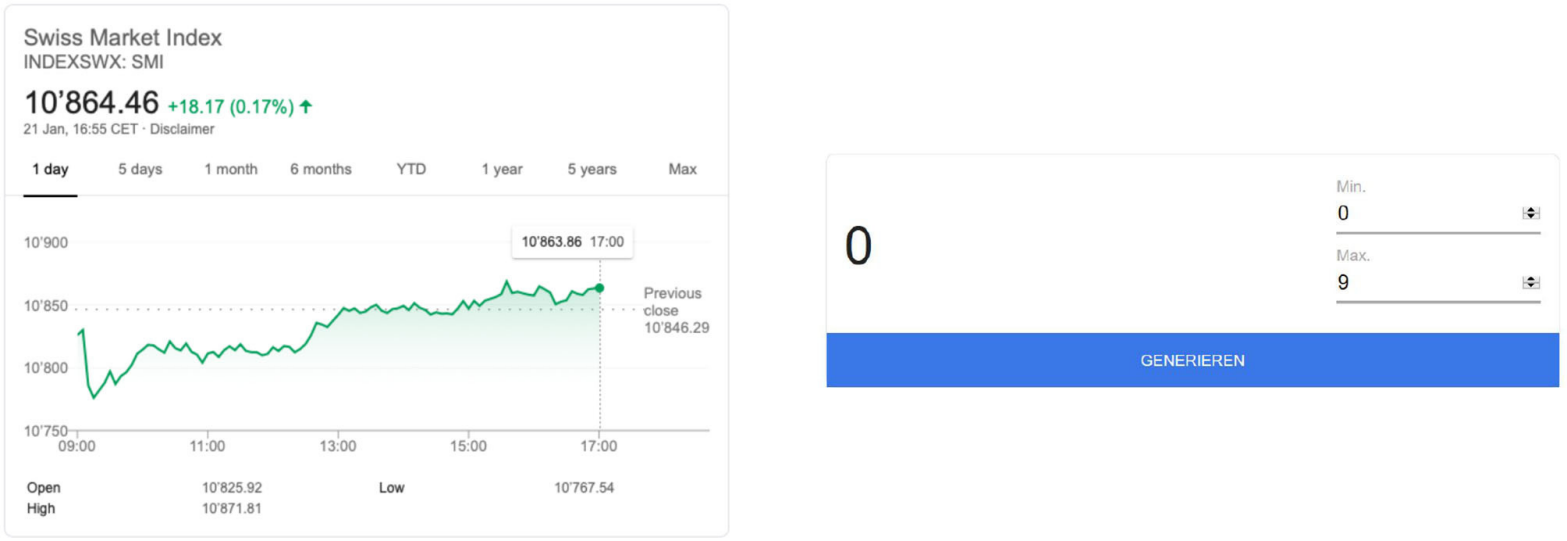
Google widget for SMI/random number.

I implement two treatments in a between-subjects design. Under both conditions, participants report outcomes using an online form. In treatment 1, the payoff is determined by the second decimal place of either the Swiss Market Index (SMI) or the DAX Performance Index (DAX) at a particular point in time; the reported value equals the payoff in CHF. Participants in the experiment are asked to lookup the value of their respective index of choice on a Google Widget, showing either the SMI or the DAX (see Figure 1 for an example of the SMI). In treatment 2, I measure dishonest behavior using a neutrally framed randomization device. The payoff is determined by looking up a random number (between zero and nine) on a Google Widget—the reported value equals the payoff in CHF. Because the payoff participants receive for participation depends on the reported value, there is a clear incentive to report higher numbers. I emphasize that I do not know about participants' choice of index/random number, lending credibility to the unobservability of the source of uncertainty, for which it is important to avoid reputation and strategic concerns.^7^ As opposed to previous studies (e.g., Cohn et al., 2014), a subject's payoff is not dependent on others' choices^8^. By the design, I cannot detect dishonesty at the individual level, but, because I know the actual distribution of values^9^ I can infer dishonesty for different subpopulations. The full set of experimental instructions can be found in the Appendix^10^.

The non-strategic nature of the experiment makes it rather easy to establish different environments in which I can hold constant important features of the decision task, while varying context in a way that can influence norms.

Subsequent to the main experiment, I examine the role of dispositional greed in explaining potential differences in dishonest behavior between the two groups. I focus on the Dispositional Greed Scale DGS (Seuntjens et al., 2015) to measure individual differences in people's propensity to be greedy. All items (e.g., “As soon as I have acquired something, I start to think about the next thing I want.”) were rated using a five-point Likert-scale, ranging from strongly disagree (1) to strongly agree (5). It has been shown that greedy people take more and contribute less in economic games (Seuntjens et al., 2015) and are more willing to accept bribes and engage in unethical behavior (Seuntjens et al., 2019). If financial markets are linked to norms that encourage greedy behavior, I expect that experimental measures of honesty will differ in these two treatments. I additionally elicit subject's risk attitudes using the survey questions developed by Dohmen et al. (2011).

### 2.2. Procedures

I recruited participants from the participant pool of the behavioral lab at the University of St.Gallen. This allows us to attentively control the pool of participants, which mitigates experimenter control problems. Additionally, the design is conceptually rather simple, which should reduce concerns about participants' mental performance being worse in the online setting compared to a laboratory setting^11^. Because the design requires that I conduct sessions during the trading hours of the SMI and DAX stock indices, participants were asked to select a time slot before taking part in the study. The link to the study was sent out in a separate e-mail shortly before the session started. Due to the nature of the experiments, some participants could access the experiment from their home, while others could do so in a noisy environment. I thus asked participants to make sure that they are in a quiet place without any distractions when starting the experiment.

In the experiment, I informed participants that the data is anonymized and treated confidentially. The context of the experiment was framed as a survey on health-related and and risk-related questions, for which participants are being paid. Participants first received instructions of the experiment via the experimental software oTree (Chen et al., 2016). The experiment then proceeds to the game, and participants were assigned to one of the conditions, assuring equal distribution of treatments within one experimental session.

Participants were compensated with a fixed participation fee of CHF 6 plus an additional payoff that varied with each participant and was conditional on a random draw (i.e., ranging between CHF 0 and CHF 9). Payments were sent to the participants' bank accounts the evening of the day of participation. To strengthen the credibility of the payment procedure, I asked subjects to enter their bank account information that is (or will be) associated with their PayPal account in the description of the study as well as in the experimental instructions. I asked participants for their bank information on a separate website connected to a separate database that I cannot link with the experimental data. This also reduces the possibility that some subjects will participate more than once. The average duration of an experimental session was about 9 min.

## 3. Results

### 3.1. Summary Statistics

The participants in the study were 135 students at the University of St. Gallen and the Fachhochschule St. Gallen. In terms of gender, the sample is quite balanced. The sample includes 69 (0.51) men and 66 (0.49) women with an average age of 23.97 years, ranging from 19 to 51. Table 1 provides summary statistics^12^.

**Table 1:** Summary statistics by group.

**Variable**	**Levels**	**n**	**Min**	$\bar{\text{x}}$	**Max**
Age (in years)	*T*_*RN*_	67	18	24.37	51
	*T*_*FM*_	68	19	23.57	30
*p* = 0.67	all	135	18	23.97	51
Gender	*T*_*RN*_	67	0	0.48	1
	*T*_*FM*_	68	0	0.50	1
*p* = 0.80	all	135	0	0.49	1
Income	*T*_*RN*_	67	0	0.43	1
	*T*_*FM*_	68	0	0.59	1
*p* = 0.07	all	135	0	0.51	1
Income source	*T*_*RN*_	67	0	0.55	1
	*T*_*FM*_	68	0	0.56	1
*p* = 0.94	all	135	0	0.56	1
Risk aversion	*T*_*RN*_	67	2	6.04	10
	*T*_*FM*_	68	1	5.82	10
*p* = 0.37	all	135	1	5.93	10

Regarding the categorial variables, about 51 percent of respondents reported to have between CHF 500 and CHF 1,499 at their disposal per month. When asked about their sources of income, more than 50 percent of respondents indicated their family, 30 percent referenced their job, and 10 percent reported a scholarship as their main source of income. Sixty-seven percent of participants ranked their health status as excellent or very good. Table A1 in the Appendix provides further details on the subjects' demographics across different samples.

### 3.2. Main Results

**Figure 2:**
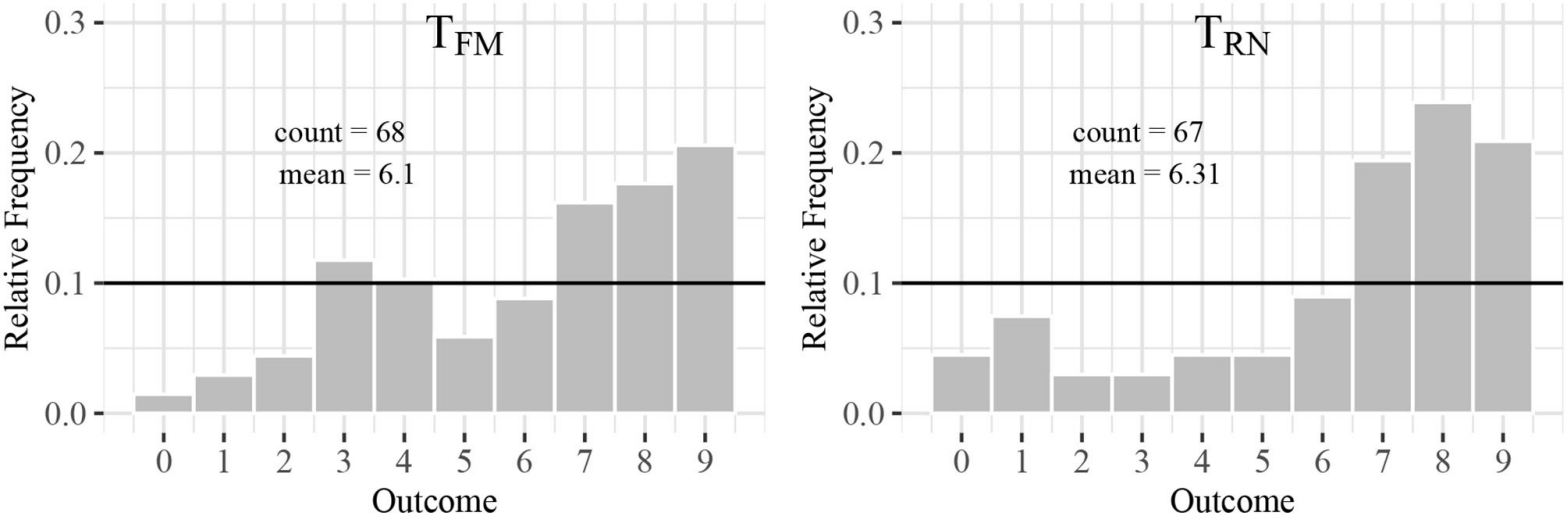
Reports of random draws. The figures show reported random draws conditional on treatment assignment.

Figure 2 shows the distribution of reported outcomes conditional on treatment assignment, and Table 2 shows estimates of treatment effects on reported outcomes (OLS) and reporting very high outcomes (Probit). In general, numbers above (below) six are significantly more (less) frequently reported than their expected true share of 10 percent (*p* < 0.001). This suggests that some participants reported higher numbers than the one they had actually seen. I can thus confirm the findings on dishonest behavior from previous studies (Fischbacher and Föllmi-Heusi, 2013). Contrary to the expectations, participants in treatment *T*_*FM*_ do not cheat more frequently than participants in treatment *T*_*RN*_ (Kolmogorov-Smirnov test: *p* = 0.896). I further observe that controlling for additional individual characteristics does not have an effect on the significance of the differences between the two treatments. This confirms previous studies, which find no significant differences in student samples between a financial and a neutral context (Huber and Huber, 2020). Similarly, Cappelen et al. (2013) do not find a significant effect when priming students to think about markets.^13^. I consider additional heterogeneous treatment effects. As previous research shows (Capraro, 2018; Gerlach et al., 2019), I find that women are more honest on average (*p* = 0.041).

**Table 2:** Results of OLS and probit regressions.

	**Value reported** **(1)**	**High report** **(2)**
*T*_*FM*_	−0.094	−0.089
	(0.452)	(0.085)
Gender	−0.930^**^	−0.124
	(0.451)	(0.084)
Constant	5.064^***^	
	(1.570)	
Controls	Yes	Yes
Observations	135	135
R^2^	0.046	
Adjusted R^2^	0.016	

*The table shows (1) OLS estimates of the treatment effects on the reported random draw defined on the interval between 0 and 9 and (2) probit estimates of the treatment effects of reporting very high values (i.e., > 6). The reference category is T_RN_. Additional independent variables include age in years, dummies for being female, different study major*.

*** * Significant at the 1 percent level*.

** Significant at the 5 percent level. * Significant at the 10 percent level.

I further examine the role of dispositional greed in explaining potential differences in dishonest behavior between the two groups. It is possible that the financial market setting evokes greedy behavior. Earlier research shows that greed is associated with fraudulent behavior (Seuntjens et al., 2019). However, I cannot find a significant impact of dispositional greed on dishonest behavior (*p* = 0.841).

### 3.3. Elicitation of Descriptive Norms

A large body of research shows that dishonest behavior also depends on the social norms implied by the dishonesty of others or by beliefs about what constitutes honest behavior (Fischbacher and Föllmi-Heusi, 2013; Cohn et al., 2014; Kocher et al., 2018). To identify norms separately from behavior, I use the norm elicitation method by Krupka and Weber (2013). In particular, I aim to test whether different expectations exist toward dishonest behavior when the source of uncertainty is a stock market index. I thus aim to test whether different expectations exist toward dishonest behavior when the source of uncertainty is a stock market index. The focus in this research is on descriptive norms.

**Figure 3:**
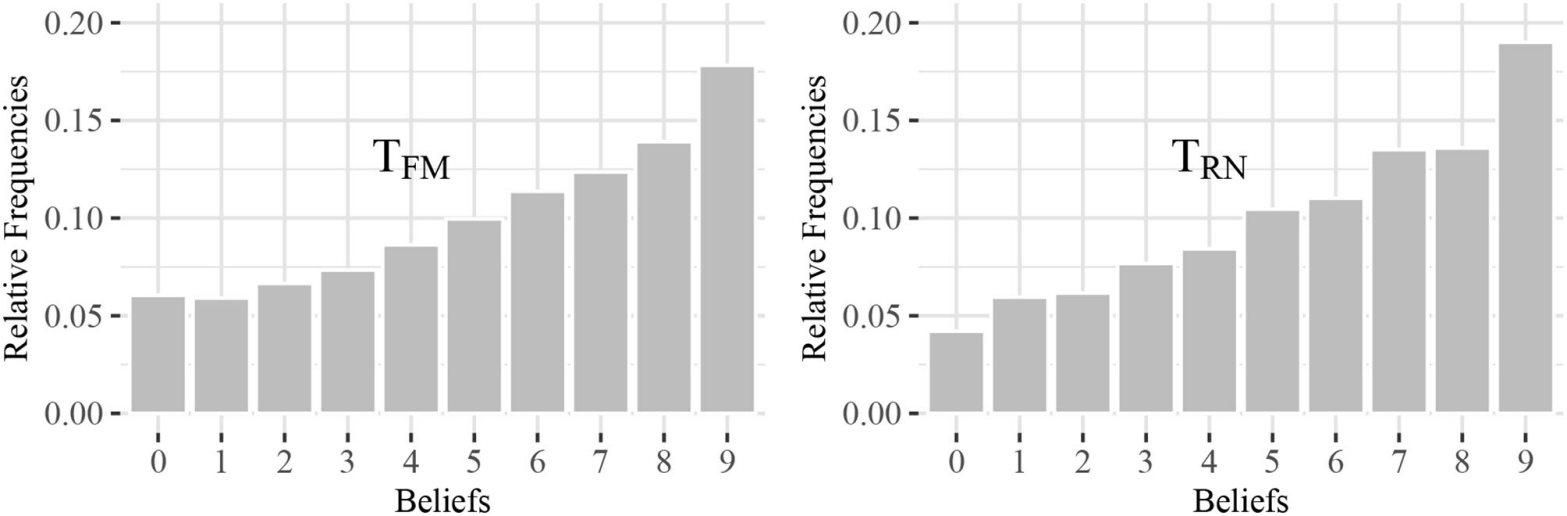
Beliefs about reported payoffs in reference experiment. The figures show the beliefs about reported payoffs in a reference experiment in terms of the share of participants that reported a payoff.

I conduct an additional experiment with a new set of subjects. In the experiment, participants must guess other participants' reporting behavior. More specifically, I prompt participants to predict the behavior of other participants in a previously run experiment (i.e., the “reference experiment”). On the first page of the experiment, I explain the setting of the reference experiment. Participants then guess what percentage of participants reported a specific payoff. They were paid depending on the accuracy of their predictions (Fischbacher and Föllmi-Heusi, 2013). Participants could earn CHF 9 if they guess all shares correctly. For every percentage point deviation from the correct share, I reduce participants' payoff by CHF 0.1. The minimum payoff in the belief elicitation task is CHF 1. Participants received a show-up fee of CHF 6 that was added to the earnings from the experiment. I recruited participants from the participant pool of the behavioral lab at the University of St.Gallen and excluded all subjects with previous experience in similar experiments. In total, 95 participants took part in this experiment (48 had to guess the behavior in *T*_*FM*_ and 47 in *T*_*RN*_). Figure 3 shows participants' beliefs about the behavior of others in terms of honesty. The data shows that beliefs increase in the reported number. Subjects report a belief that a fraction of more than 10 percent reported the highest number. Thus, they believe that similar decision makers act dishonestly. I observe that the distributions of beliefs correspond fairly closely to the distributions of the actual reporting behavior. This shows that participants act in accordance with the perceived norm. I do not find a significant influence of the frame-specific device on beliefs. A Kolmogorov-Smirnov test indicates that the two distributions are not significantly different from each other (*p* = 0.880). More specifically, the frame-specific device does not shift participants' beliefs about the prevailing honesty norm.

### 3.4. Laboratory Evidence

I extend the study to the traditional laboratory (using the same treatment conditions *T*_*RN*_ and *T*_*FM*_) in order to understand whether the environment (i.e., traditional lab experiment or online experiment) has an effect on the decision to be honest. To make online experiments comparable with laboratory experiments, investigating potential differences in results is of crucial importance. The environment of subjects in a laboratory is quite different from the environment of subjects taking part in the study using a Web browser at home.^14^. It is possible that participants feel more socially distant from others in an online environment. This might reduce participants' need to adhere to behavior norms. In a typical laboratory setting, participants can see each other and possibly even talk to each other. I therefore replicate the experiment in the more constrained setting of the traditional physical laboratory.

I recruited participants from the participant pool of the behavioral lab at the University of St.Gallen using the same instructions (i.e., random number or financial market), the same incentive-compatible design, and the same decision interface. This ensures the credibility of comparability of the two groups. During the experiment, each participant sat at a randomly assigned, separated PC terminal. No form of communication was allowed during the experiment. I conducted all sessions at the behavioral lab in St. Gallen. I excluded all subjects with previous experience in the honesty task. The participants in the study were 135 students at the University of St. Gallen and the Fachhochschule St. Gallen. The sample appears balanced across treatment conditions (see Table A2 in the Appendix). This is expected due to the randomized assignment to treatment. To make payments in the lab as salient as in the online setting, payments were sent to the participants' bank accounts the evening of the day of participation.

**Figure 4:**
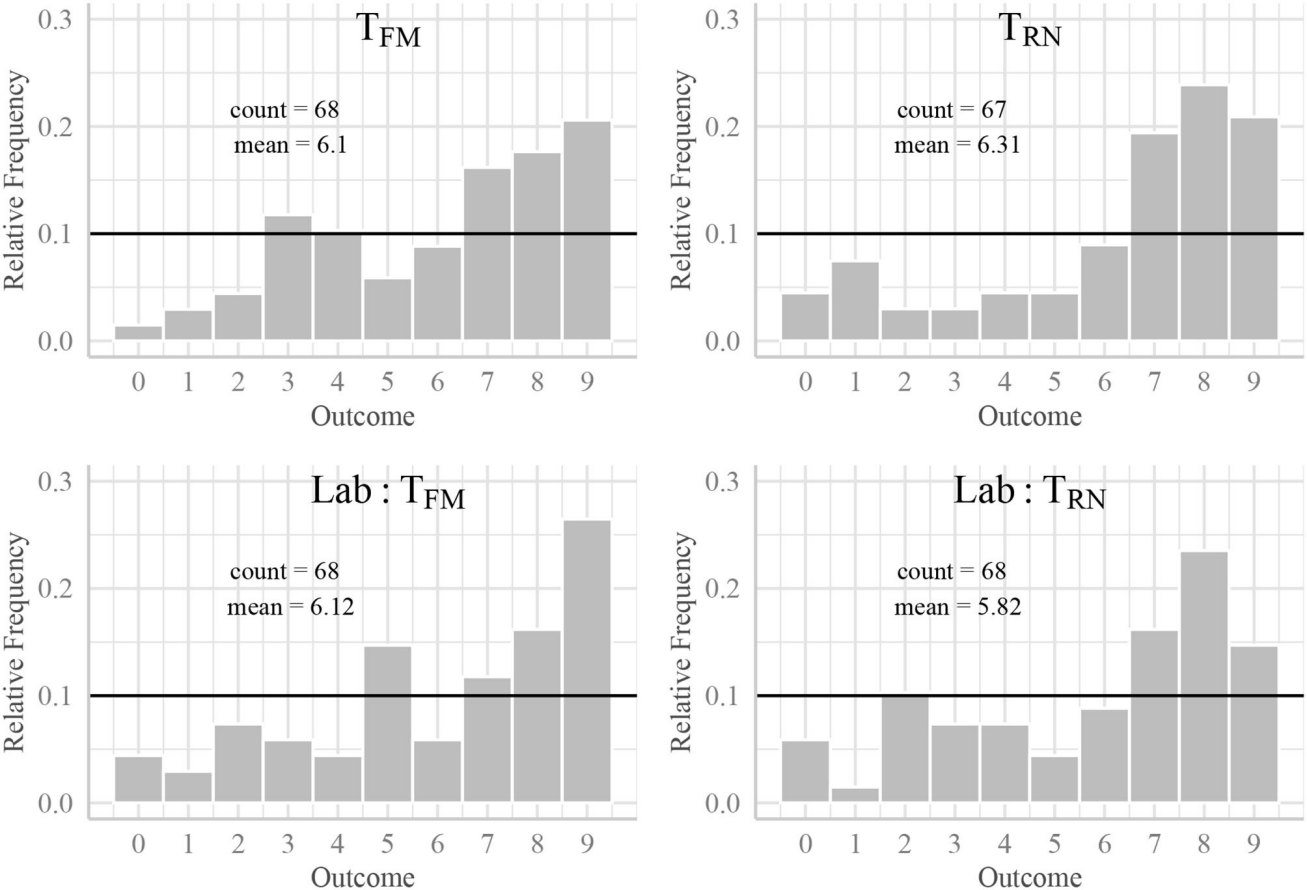
Reports of random draws. The figures show reported random draws conditional on treatment assignment.

Figure 4 shows the distribution of reported outcomes conditional on treatment assignment. As a means of comparison, I show both results from the online and the laboratory experiment. Supporting earlier results from the online setting, I find that participants in the lab are not more dishonest in treatment *T*_*FM*_. To put it differently, mean outcomes do not significantly differ depending on the environment (KS test *p* = 0.734).

In a next step, I explore differences in decision times depending on the environment (i.e., laboratory or online). A large body of literature suggests that deception is cognitively more demanding than responding honestly, and, thus, honesty is considered as behavioral default (Foerster et al., 2013). This conclusion was supported by more recent studies which find that time pressure promotes honesty (Capraro, 2017; Capraro et al., 2019). Other research, however, reported the opposite (Shalvi et al., 2012). More precisely, Shalvi et al. (2012) show that lying is an initial, automatic tendency that is overcome only if sufficient time to deliberate is available and if dishonest behavior cannot be justified. This is supported by earlier findings in neuropsychological research showing that the right dorsolateral prefrontal cortex, a brain area involved in executive control, is associated with overriding selfish impulses in economic decisions (Knoch et al., 2006) and that this area, together with two other brain areas associated with self-control, is activated when individuals make an effort to forgo lying (Greene and Paxton, 2009).

To control for differences in decision times, I observe the time difference (in seconds) between the instruction to look up the stock index value and the actual reporting of results (this is not visible to participants). I find that the average decision time in the laboratory is significantly higher than the average decision time online (*p* = 0.042). This confirms previous results (Anderhub et al., 2001; Hergueux and Jacquemet, 2015). I include decision times in the regressions presented in Table 3, both as an additional control variable and as an interaction term with a dummy variable indicating whether the experiment was conducted in the lab or online. This allows capturing the variation in honest behavior that is induced by the environment and decision times. The results confirm what (Shalvi et al., 2012) had indicated—participants who take longer to report are more honest. However, this is only true for laboratory subjects. As depicted in Table 3, a 1-s increase in the time to report changes the report by –0.034 (*p* = 0.042) for participants in the laboratory. This could potentially be explained by the fact that the latter group of participants take more time to think about others' behavior (i.e., what is socially acceptable).

**Table 3:** Results of OLS regressions.

	**Reporting time**
Seconds	0.365
	(0.457)
Lab	0.007
	(0.013)
Seconds:Lab	−0.034^**^
	(0.017)
Constant	5.863^***^
	(1.375)
Controls	Yes
Observations	270
R^2^	0.028
Adjusted R^2^	0.010

*The table shows OLS estimates of reporting time (i.e., time spent on the reporting page) as well as its interaction with a dummy variable indicating whether subjects conducted the study in the laboratory or at home on the reported random draw defined on the interval between 0 and 9*.

*** *Significant at the 1 percent level*.

** Significant at the 5 percent level. * Significant at the 10 percent level.

My conclusion that honesty is deliberative should, however, be interpreted with caution. A recent replication study (Van der Cruyssen et al., 2020) was not able to yield support for the original study of Shalvi et al. (2012). Having said this, my results are in line with a recent meta-analysis, indicating that, honesty is deliberative, but only when no concrete other is harmed (Köbis et al., 2019).

Finally, I look at potential gender differences in terms of reporting behavior in the laboratory experiment by including a dummy variable for gender into the regression. I additionally include an interaction between the gender dummy and a dummy indicating whether subjects conducted the study in the laboratory or online. This interaction term allows testing whether either gender is more sensitive to the environment. Table A3 in the Appendix presents the results. The results illustrate that the coefficient of the gender dummy is significantly different depending on the environment—men are significantly more honest when they conduct the experiment in the laboratory as opposed to online (*p* = 0.021). Thus, lesser social distance affects truth-telling behavior of men. Another explanation may be subjects' reputation. Even though the experimental design allows to credibly eliminate any reputation concerns, it is possible that participants feel observed by other students (and the experimenter) when they are sitting in the lab. Even the slightest cues of being observed seem to affect male but not female reporting behavior.

## 4. Conclusion

In this study, I investigate dishonest behavior using frame-specific randomization devices to vary the situational context of the game (i.e., stock market or neutral context). The results show no significant differences in dishonest behavior between the two groups. This indicates that this specific source of uncertainty is not prone to the social norm effects documented in the literature. The findings confirm previous studies (Cappelen et al., 2013; Huber and Huber, 2020) and extend them by varying the setting.

As different environments can render different social norms salient, I replicate the experiment in the more constrained setting of the traditional physical laboratory. I cannot confirm significant differences in dishonest behavior depending on the environment. Additional estimations capture the variation in honest behavior that is induced not only by the environment, but also decision times. I find that participants who take longer to report are more honest. However, this is only true for subjects in the physical laboratory. Depending on the experimental setting, the inclusion of controls for differences in decision times among online subjects can be important for future studies. Finally, the results suggest that even the slightest cues of being observed affects truth-telling behavior of male but not female participants.

The present study has some limitations. First, the frame-specific device showing a stock market index may not have been strong enough to activate the norms related to financial markets. Second, due to the nature of the online experiment, some participants may have accessed the experiment from a quiet place, while others may have done so in a noisy environment. However, the design is conceptually rather simple, which should reduce concerns that subjects are less attentive in the online environment.

Lastly, this paper also makes a methodological contribution. The experimental approach to measure dishonest behavior outside of the lab can be applied broadly in decentral experimental setups as well as surveys. Non-physical and verifiable sources of uncertainty are key to extending the valid measurement of dishonest behavior to broader settings such as online experimentation. The non-strategic nature of the experiment makes it rather easy to establish different environments in which one can hold constant important features of the decision task—the payoffs, the description of the way the task works, and so on—, while varying context in a way that can influence social norms.

## Data Availability Statement

The raw data supporting the conclusions of this article will be made available by the authors, without undue reservation.

## Ethics Statement

The study was reviewed and approved by the Ethics Committee of the University of St. Gallen, Switzerland (HSG-EC-20210715-A). The patients/participants provided their written informed consent to participate in this study.

## Author Contributions

The author confirms being the sole contributor of this work and has approved it for publication.

## Conflict of Interest

The author declares that the research was conducted in the absence of any commercial or financial relationships that could be construed as a potential conflict of interest.

## Publisher's Note

All claims expressed in this article are solely those of the authors and do not necessarily represent those of their affiliated organizations, or those of the publisher, the editors and the reviewers. Any product that may be evaluated in this article, or claim that may be made by its manufacturer, is not guaranteed or endorsed by the publisher.

## Footnotes

^1^Capraro and Perc (2021) provide an exhaustive review of moral and norm framing effects.

^2^Earlier studies indicate that a lower degree of social distance between parties increases prosocial behavior (Hoffman et al., 1996; Bohnet and Frey, 1999; Charness et al., 2007; Charness and Gneezy, 2008).

^3^The framing effect concept was coined by Tversky and Kahneman (1981). This study focuses on framing effects related to the labeling of the situational context of the experiment.

^4^In the jurisdiction of the original study (Cohn et al., 2014), the banking industry has a very bad reputation at the time of the experiment. They are perceived to be less honest than doctors, to be less honest than the general population and to behave about as dishonestly as prison inmates. This does not apply to other jurisdictions in Rahwan et al. (2019).

^5^Pfister et al. (2019) find that dishonest behavior does not only entail aforementioned intrinsic costs but that they also come with cognitive costs that emerge right before and while a person deliberately violates a rule.

^6^Fischbacher and Föllmi-Heusi (2013) had participants roll a die *in private* and report their roll. Participants were paid CHF 1, 2, 3, 4, and 5 for the corresponding outcome and CHF 0 for an outcome of 6.

^7^To test whether fear of detection plays a role, I elicit subjects' risk attitudes to test whether there is a relationship between risk aversion and dishonest behavior. If the fear of detection is a relevant issue in the design, I expect that more risk averse subjects are less dishonest. I find that subjects' risk aversion does not significantly decrease reported values (*p* = 0.519). It can thus be concluded that punishment concerns do not play a role in the experimental design.

^8^As pointed out by Stöckl (2015), due to the competitive aspect of the experiment in Cohn et al. (2014), subjects actually play a strategic game.

^9^The distribution of second decimals in the range of [0,9] of the DAX and SMI stock index is not only known approximately but exactly, as we look up the data in 5-min intervals at a later point in time in the respective Google Widgets. It is important to emphasize that we are not able to observe the same data as participants during the time of the experiment. I show in Figure A1 of the Appendix that the values are indeed equally distributed.

^10^On a cautionary note, I should mention that while the two different randomizer layouts reduce participants' beliefs of experimenter-induced influence of outcomes, it should not be ignored that such differences in the specific layouts may give rise to systematic variation between conditions.

^11^Anderhub et al. (2001) show that subjects are less attentive in an online experiment compared to a class experiment. In contrast, Bosch-Domenech et al. (2002) find that the results of a guessing game are similar in both settings.

^12^For ease of interpretation, I recoded the categorial variables into binary variables (i.e., dummy coding). Gender is a dummy variable that takes on the value 1 when the participant is female. Income is a dummy variable that takes on the value of 1 if the participant has between CHF 500 to 1,499 at his/her disposal per month. Income source is a dummy taking on the value of 1 if their main source of income is their family.

^13^In the base treatment, participants are asked to write about the city of Bergen, Norway. In the market treatment, they are asked to write about when they had benefited from buying or selling a good or service.

^14^Previous studies that compare laboratory to Internet data mostly use a very different subject pool in the online experiment. I, however, conduct an online experiment with subjects comparable to the subjects in the laboratory, as I recruit subjects from the same participant pool.

## References

[B1] Abeler J. Becker A. Falk A. (2014). Representative evidence on lying costs. J. Pub. Econ. 113, 96–104. 10.1016/j.jpubeco.2014.01.005

[B2] Anderhub V. Müller R. Schmidt C. (2001). Design and evaluation of an economic experiment via the internet. J. Econ. Behav. Organ. 46, 227–247. 10.1016/S0167-2681(01)00195-0

[B3] Andreoni J. (1995). Warm-glow versus cold-prickle: the effects of positive and negative framing on cooperation in experiments. Qu. J. Econ. 110, 1–21.

[B4] Barron K. (2019). Lying to Appear Honest. WZB Discussion Paper No. SP II.

[B5] Bohnet I. Frey B. S. (1999). The sound of silence in prisoner's dilemma and dictator games. J. Econ. Behav. Organ. 38, 43–57.

[B6] Bosch-Domènech A. Montalvo J. G. Nagel R. Satorra A. (2002). One, two, (three), infinity,…: newspaper and lab beauty-contest experiments. Am. Econ. Rev. 92, 1687–1701. 10.1257/000282802762024737

[B7] Cappelen A. W. Sørensen E. Ø. Tungodden B. (2013). When do we lie? J. Econ. Behav. Organ. 93, 258–265. 10.1016/j.jebo.2013.03.037

[B8] Capraro V. (2017). Does the truth come naturally? Time pressure increases honesty in one-shot deception games. Econ. Lett. 158, 54–57. 10.1016/j.econlet.2017.06.015

[B9] Capraro V. (2018). Gender differences in lying in sender-receiver games: a meta-analysis. Judg. Decis. Making 13, 345–355. 10.2139/ssrn.2930944

[B10] Capraro V. Perc M. (2021). Mathematical foundations of moral preferences. J. R. Soc. Interf. 18. 10.1098/rsif.2020.0880 PMC8086879 33561377

[B11] Capraro V. Schulz J. Rand D. G. (2019). Time pressure and honesty in a deception game. J. Behav. Exper. Econ. 79, 93–99. 10.1016/j.socec.2019.01.007

[B12] Capraro V. Vanzo A. (2019). The power of moral words: Loaded language generates framing effects in the extreme dictator game. Judg. Decis. Making 14, 309–317. 10.2139/ssrn.3186134

[B13] Chang D. Chen R. Krupka E. (2019). Rhetoric matters: a social norms explanation for the anomaly of framing. Games Econ. Behav. 116, 158–178. 10.1016/j.geb.2019.04.011

[B14] Charness G. Gneezy U. (2008). What's in a name? Anonymity and social distance in dictator and ultimatum games. J. Econ. Behav. Organ. 68, 29–35. 10.1016/j.jebo.2008.03.001

[B15] Charness G. Haruvy E. Sonsino D. (2007). Social distance and reciprocity: an Internet experiment. J. Econ. Behav. Organ. 63, 88–103. 10.1016/j.jebo.2005.04.021

[B16] Chen D. L. Schonger M. Wickens C. (2016). oTree—An open-source platform for laboratory, online, and field experiments. J. Behav. Exper. Finance 9, 88–97. 10.1016/j.jbef.2015.12.001

[B17] Cohn A. Fehr E. Maréchal M. A. (2014). Business culture and dishonesty in the banking industry. Nature 516, 86–89. 10.1038/nature13977 25409154

[B18] Dohmen T. Falk A. Huffman D. Sunde U. Schupp J. Wagner G. G. (2011). Individual risk attitudes: measurement, determinants, and behavioral consequences. J. Eur. Econ. Assoc. 9, 522–550. 10.1111/j.1542-4774.2011.01015.x

[B19] Dreber A. Ellingsen T. Johannesson M. Rand D. G. (2013). Do people care about social context? Framing effects in dictator games. Exper. Econ. 16, 349–371. 10.1007/s10683-012-9341-9

[B20] Dufwenberg M. Gächter S. Hennig-Schmidt H. (2011). The framing of games and the psychology of play. Games Econ. Behav. 73, 459–478. 10.1016/j.geb.2011.02.003

[B21] Ellingsen T. Johannesson M. Mollerstrom J. Munkhammar S. (2012). Social framing effects: preferences or beliefs? Games Econ. Behav. 76, 117–130. 10.1016/j.geb.2012.05.007

[B22] Erat S. Gneezy U. (2012). White lies. Manag. Sci. 58, 723–733. 10.1287/mnsc.1110.1449

[B23] FBI (2020). Insurance Fraud. Availabel online at: https://www.fbi.gov/stats-services/publications/insurance-fraud

[B24] Fehr E. Schmidt K. M. (2006). The economics of fairness, reciprocity and altruism–experimental evidence and new theories. Handbook Econ. Giving Altruism Reciprocity 1, 615–691. 10.1016/S1574-0714(06)01008-6

[B25] Fischbacher U. Föllmi-Heusi F. (2013). Lies in disguise-an experimental study on cheating. J. Eur. Econ. Assoc. 11, 525–547. 10.1111/jeea.12014

[B26] Foerster A. Pfister R. Schmidts C. Dignath D. Kunde W. (2013). Honesty saves time (and justifications). Front. Psychol. 4:473. 10.3389/fpsyg.2013.00473 23888151 PMC3719030

[B27] Gerlach P. Teodorescu K. Hertwig R. (2019). The truth about lies: a meta-analysis on dishonest behavior. Psychol. Bull. 145, 1–44. 10.1037/bul0000174 30596431

[B28] Gneezy U. (2005). Deception: the role of consequences. Am. Econ. Rev. 95, 384–393. 10.1257/0002828053828662

[B29] Gneezy U. Kajackaite A. Sobel J. (2018). Lying aversion and the size of the lie. Am. Econ. Rev. 108, 419–453. 10.1257/aer.20161553

[B30] Greene J. D. Paxton J. M. (2009). Patterns of neural activity associated with honest and dishonest moral decisions. Proc. Natl. Acad. Sci. U.S.A. 106, 12506–12511. 10.1073/pnas.0900152106 19622733 PMC2718383

[B31] Henrich J. Boyd R. Bowles S. Camerer C. Fehr E. Gintis H.. (2001). In search of homo economicus: behavioral experiments in 15 small-scale societies. Am. Econ. Rev. 91, 73–78. 10.1257/aer.91.2.73

[B32] Hergueux J. Jacquemet N. (2015). Social preferences in the online laboratory: a randomized experiment. Exper. Econ. 18, 251–283. 10.1007/s10683-014-9400-5

[B33] Hoffman E. McCabe K. Smith V. L. (1996). Social distance and other-regarding behavior in dictator games. Am. Econ. Assoc. 86, 653–660.

[B34] Huber C. Huber J. (2020). Bad bankers no more? Truth-telling and (dis)honesty in the finance industry. J. Econ. Behav. Organ. 180, 472–493. 10.1016/j.jebo.2020.10.020

[B35] Internal Revenue Service (2019). Federal Tax Compliance Research: Tax Gap Estimates for Tax Years 2011–2013. Publication 1415.

[B36] Kay A. C. Ross L. (2003). The perceptual push: the interplay of implicit cues and explicit situational construals on behavioral intentions in the prisoner's dilemma. J. Exper. Soc. Psychol. 39, 634–643. 10.1016/S0022-1031(03)00057-X

[B37] Knoch D. Pascual-Leone A. Meyer K. Treyer T. Fehr E. (2006). Diminishing reciprocal fairness by disrupting the right prefrontal cortex. Science 314, 829–932. 10.1126/science.1129156 17023614

[B38] Köbis N. C. Verschuere B. Bereby-Meyer Y. Rand D. Shalvi S. (2019). Intuitive honesty versus dishonesty: meta-analytic evidence. Perspect. Psychol. Sci. 14, 778–796. 10.1177/1745691619851778 31291557

[B39] Kocher M. G. Schudy S. Spantig L. (2018). I lie? we lie! why? Experimental evidence on a dishonesty shift in groups. Manag. Sci. 64, 3995–4008. 10.1287/mnsc.2017.2800

[B40] Krupka E. L. Weber R. A. (2013). Identifying social norms using coordination games: why does dictator game sharing vary? J. Eur. Econ. Assoc. 11, 495–524. 10.1111/jeea.12006

[B41] Liberman V. Samuels S. M. Ross L. (2004). The name of the game: predictive power of reputations versus situational labels in determining Prisoner's Dilemma game moves. Person. Soc. Psychol. Bull. 30, 1175–1185. 10.1177/0146167204264004 15359020

[B42] Mazar N. Amir O. Ariely D. (2008). The dishonesty of honest people: a theory of self-concept maintenance. J. Market. Res. 45, 633–644. 10.1509/jmkr.45.6.633

[B43] Pfister R. Wirth R. Weller L. Foerster A. Schwarz K. A. (2019). Taking shortcuts: cognitive conflict during motivated rule-breaking. J. Econ. Psychol. 71, 138–147. 10.1016/j.joep.2018.06.005

[B44] Rahwan Z. Yoeli E. Fasolo B. (2019). Heterogeneity in banker culture and its influence on dishonesty. Nature 575, 345–349. 31723285 10.1038/s41586-019-1741-y

[B45] Seuntjens T. G. Zeelenberg M. Van De Ven N. Breugelmans S. M. (2015). Dispositional greed. J. Person. Soc. Psychol. 108, 917–133. 10.1037/pspp0000031 25664899

[B46] Seuntjens T. G. Zeelenberg M. Van De Ven N. Breugelmans S. M. (2019). Greedy bastards: testing the relationship between wanting more and unethical behavior. Person. Indiv. Diff. 138, 147–156. 10.1016/j.paid.2018.09.027

[B47] Shalvi S. Eldar O. Bereby-Meyer Y. (2012). Honesty requires time (and lack of justifications). Psychol. Sci. 23, 1264–1270. 10.1177/0956797612443835 22972904

[B48] Stöckl T. (2015). Dishonest or professional behavior? Can we tell? A comment on: Cohn et al. 2014, Nature 516, 86-89, “Business culture and dishonesty in the banking industry”. J. Behav. Exper. Finance 8, 64–67. 10.1016/j.jbef.2015.10.003 25409154

[B49] Tversky A. Kahneman D. (1981). The framing of decisions and the psychology of choice. Science 211, 453–458. 7455683 10.1126/science.7455683

[B50] Van der Cruyssen I. D'hondt J. Meijer E. Verschuere B. (2020). Does honesty require time? Two preregistered direct replications of experiment 2 of Shalvi, Eldar, and Bereby-Meyer (2012). Psychol. Sci. 31, 460–467. 10.1177/0956797620903716 32156182 PMC7168803

[B51] Vohs K. D. (2015). Money priming can change people's thoughts, feelings, motivations, and behaviors. J. Exper. Psychol. Gen. 144, e86–e93. 10.1037/xge0000091 26214169

[B52] Vohs K. D. Mead N. L. Goode M. R. (2008). Merely activating the concept of money changes personal and interpersonal behavior. Curr. Direct. Psychol. Sci. 17, 208–212. 10.1111/j.1467-8721.2008.00576.x

[B53] Vranka M. A. Houdek P. (2015). Many faces of bankers' identity: How (not) to study dishonesty. Front. Psychol. 6:302. 10.3389/fpsyg.2015.00302 25852616 PMC4364084

