# Supplementary material for: Investigating Dishonesty-Does Context Matter?
Source: Front Psychol. 2021 Aug 23;12:684735. doi: 10.3389/fpsyg.2021.684735 (PMC8419446; doi:10.3389/fpsyg.2021.684735)
Supplement: Supplementary file 1 [file Table_1.pdf]

6 Appendix

6.1 Additional figures and tables

Table A1: Subject demographics

Characteristic	RN Online[a]	FM Online	RN Laboratory	FM Laboratory
**Age**	23 (22, 26)	23 (22, 25)	20 (19, 21)	20 (19, 22)
**Gender**				
0=Male	35 (52%)	34 (51%)	35 (51%)	37 (54%)
1=Female	32 (48%)	33 (49%)	33 (49%)	31 (46%)
**Income Source**				
Family	37 (55%)	37 (55%)	59 (87%)	52 (76%)
Job	21 (31%)	24 (36%)	2 (2.9%)	11 (16%)
Other	2 (3.0%)	3 (4.5%)	3 (4.4%)	2 (2.9%)
Scholarship	7 (10%)	3 (4.5%)	4 (5.9%)	3 (4.4%)
**Income**				
CHF 1,500 to 2,499	26 (39%)	10 (15%)	13 (19%)	17 (25%)
CHF 2,499 to 3,499	3 (4.5%)	4 (6.0%)	1 (1.5%)	1 (1.5%)
CHF 3,500 or more	3 (4.5%)	7 (10%)	0 (0%)	4 (5.9%)
CHF 500 to 1,499	29 (43%)	40 (60%)	40 (59%)	37 (54%)
less than CHF 500	6 (9.0%)	6 (9.0%)	14 (21%)	9 (13%)
**Major**				
Assessment	4 (6.0%)	3 (4.5%)	31 (46%)	23 (34%)
B.Admin.	16 (24%)	21 (31%)	16 (24%)	16 (24%)
Economics	6 (9.0%)	6 (9.0%)	4 (5.9%)	9 (13%)
Finance	8 (12%)	10 (15%)	1 (1.5%)	6 (8.8%)
Int. Affairs	12 (18%)	5 (7.5%)	7 (10%)	2 (2.9%)
Law	3 (4.5%)	6 (9.0%)	4 (5.9%)	4 (5.9%)
Marketing	5 (7.5%)	5 (7.5%)	4 (5.9%)	5 (7.4%)
Other	13 (19%)	11 (16%)	1 (1.5%)	3 (4.4%)
**Level**				
Assessment	4 (6.0%)	3 (4.5%)	32 (47%)	26 (38%)
Bachelor	22 (33%)	26 (39%)	26 (38%)	23 (34%)
Master	36 (54%)	32 (48%)	10 (15%)	16 (24%)
PhD	5 (7.5%)	6 (9.0%)	0 (0%)	3 (4.4%)

Characteristic	RN Online[a]	FM Online	RN Laboratory	FM Laboratory
**Risk**	6.00 (5.00, 7.00)	6.00 (4.00, 7.00)	6.00 (4.00, 7.00)	6.00 (4.00, 7.00)

[a]Data represent median and interquartile range (continuous variables) or frequency (categorical variables).

Table A2: Balancing tests

	Gender	Age (in years)	Income	Income Source	Risk aversion
	(1)	(2)	(3)	(4)	(5)
T_{FM}	−0.029	1.147**	−0.044	−0.103	0.103
	(0.086)	(0.441)	(0.086)	(0.066)	(0.321)
Constant	0.485***	20.206***	0.588***	0.868***	6.779***
	(0.061)	(0.312)	(0.060)	(0.047)	(0.227)
Mean	0.47	20.74	0.56	0.81	6.80
Std deviation	0.50	2.65	0.50	0.39	1.90
Observations	136	136	136	136	136
R^2	0.001	0.048	0.002	0.018	0.001
Adjusted R^2	−0.007	0.041	−0.005	0.010	−0.007

Notes: The table shows OLS estimates of differences between the treatment conditions and the base condition T_{RN}. The constant term represents the average values for the subsample assigned to T_{RN}. For ease of interpretation, I recoded our categorial variables into binary variables (i.e., dummy coding). Gender is a dummy variable that takes on the value 1 when the participant is female. Income is a dummy variable that takes on the value of 1 if the participant has between CHF 500 to 1,499 at his/her disposal per month. Income source is a dummy taking on the value of 1 if their main source of income is their family. *** Significant at the 1 percent level. ** Significant at the 5 percent level. * Significant at the 10 percent level.

Table A3: Results of OLS regressions

	Gender differences		
	(1)	(2)	(3)
Lab	−0.223		0.615
	(0.327)		(0.503)
Gender		0.334	1.141**
		(0.327)	(0.469)
Lab:Gender			−1.511**
			(0.652)
Constant	6.194***	5.907***	5.503***
	(0.232)	(0.237)	(1.375)
Controls	Yes	Yes	Yes
Observations	270	270	270
R^2	0.002	0.004	0.027
Adjusted R^2	−0.002	0.0002	0.008

Notes: The table shows OLS estimates of gender (0 if male, 1 if female) as well as its interaction with a dummy variable indicating whether subjects conducted the study in the laboratory or at home on the reported random draw defined on the interval between 0 and 9. Note that the reference category for the gender is male. *** Significant at the 1 percent level. ** Significant at the 5 percent level. * Significant at the 10 percent level.

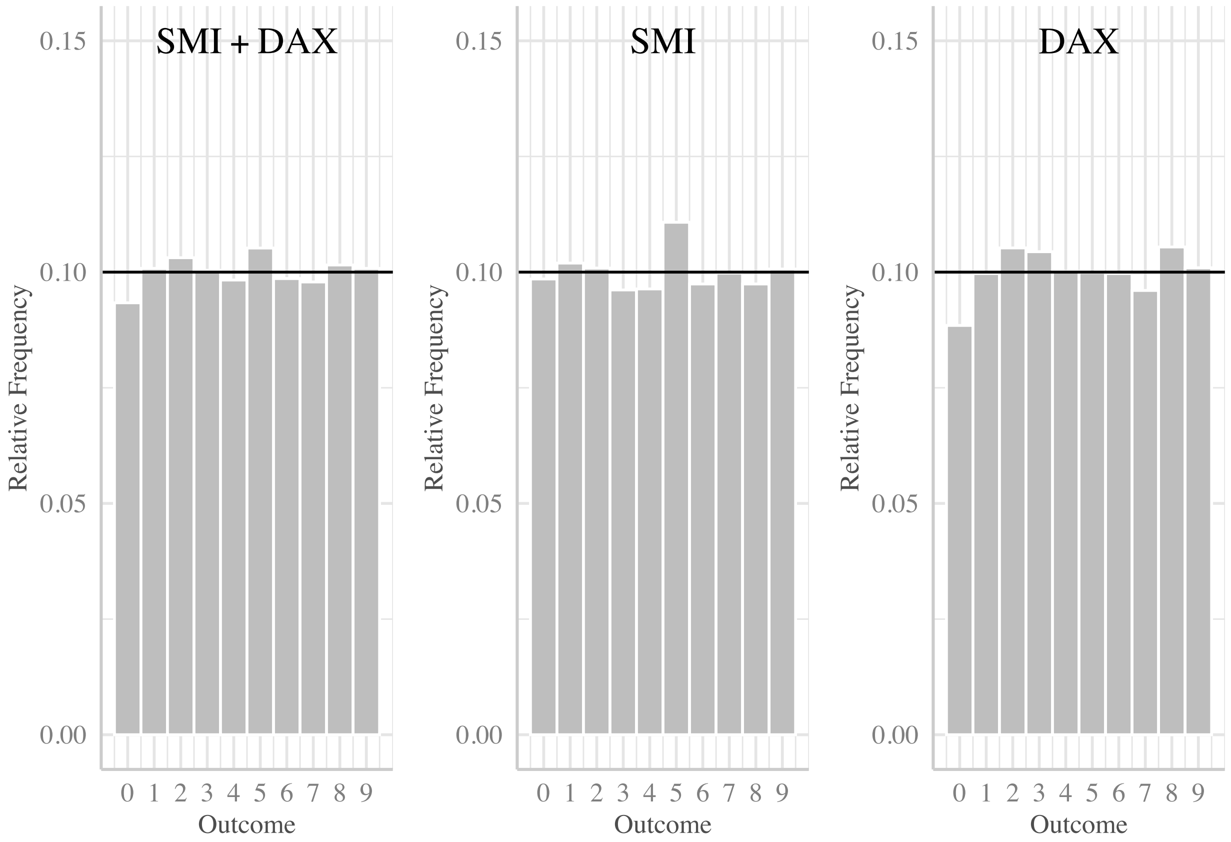

Figure A1:

Note: The figures show actually occured draws (i.e., the second decimal value) of our two natural randomization devices—the SMI and the DAX stock index value. The data has been collected via daily downloads of HTML files of the respective Google Finance Widgets, containing the actually displayed values of SMI and DAX available to the participants.

6.2 Experimental instructions

Financial market treatment

Welcome to our experiment! In this experiment I want you to answer a couple of survey questions as well as questions regarding your health insurance plan, for which you are being paid for. The questions are not meant to test you and there are no right or wrong answers. The experiment is being conducted by a research group at the University of St. Gallen. Your participation is completely voluntary. Please read the following explanations carefully.

For answering the short survey, I compensate you with a fixed participation fee of 6 CHF plus an additional payoff that is not the same for every participant. This additional payoff is determined randomly by the second decimal place of a stock market index at the point in time I ask you to look it up. You can see how much additional payoff you receive conditional on the value of the second decimal from the chart below.

Current value of second decimal digit of stock index:	0	1	2	3	4	5	6	7	8	9
Resulting additional payoff (CHF):	0.00	1.00	2.00	3.00	4.00	5.00	6.00	7.00	8.00	9.00

To determine the additional payoff, you go to the Google Widget that shows the value of either the Swiss Market Index (SMI) OR the DAX Performance Index (DAX) as presented below. You decide, which of the two indices you would like to use for this, and I will not know about your choice of the index. I will ask you to report the current value of the second decimal place of the value of the selected index (marked here with a red circle). This number changes frequently and I need you to report only the value in the particular moment when you first look at it once I have asked you to do so.

For example, if the SMI value is 10'270.80, as in the example above, the correctly reported value is 0 and thus provides an additional payoff of 0 CHF. In that case you receive a total compensation for participating in our experiment of 6 CHF (i.e., 0 CHF + 6 CHF). Remember that only the second decimal place of the selected index value should be reported.

In total you can earn between 6 CHF and 15 CHF in this experiment that takes about 25 minutes to finish. The structure of the experiment is as follows:

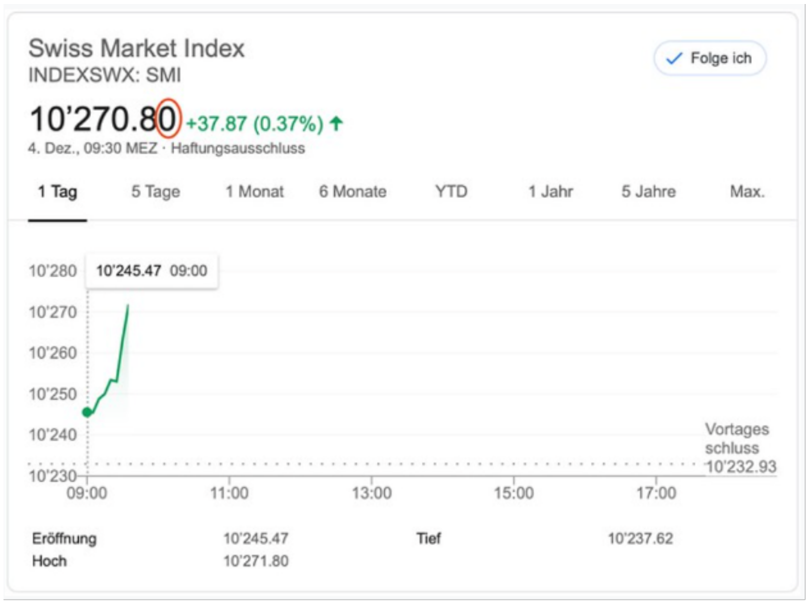

Figure A2: Google Widget for SMI

Note: Google Widget for SMI shown to participants.

1. Personal survey
2. Questions regarding your decision on health insurance
3. SMI or DAX lookup to determine additional payment
4. Two additional questionnaires

The information that I obtain will be used only for our research. It will be analyzed anonymously and will not be disclosed to third parties. The responses that you give cannot be connected to your identity by design of the experiment.

If at any time or for any reason you decide not to participate anymore, you are free to stop the experiment whether you have started or not.

Please make sure that you are in a quiet place without any distractions.

The payment for your participation will be sent to your bank account and I will ask you for your bank information on a separate website. Note that the money can only be transferred to a Swiss bank account, a PayPal or a Revolut account. Please have your IBAN or your PayPal e-mail address ready. Please note also that it is not possible for us to link your bank account information to the responses you give in this experiment as they are stored in a different database.

Please start this experiment only on your PC/mac and not on a mobile device.

Random number treatment

Welcome to our experiment! In this experiment I want you to answer a couple of survey questions as well as questions regarding your health insurance plan, for which you are being paid for. The questions are not meant to test you and there are no right or wrong answers. The experiment is being conducted by a research group at the University of St. Gallen. Your participation is completely voluntary. Please read the following explanations carefully.

For answering the short survey, I compensate you with a fixed participation fee of 6 CHF plus an additional payoff that is not the same for every participant. This additional payoff is determined randomly by a random number. You can see how much additional payoff you receive conditional on the generated random number.

Random number:	0	1	2	3	4	5	6	7	8	9
Resulting additional payoff (CHF):	0.00	1.00	2.00	3.00	4.00	5.00	6.00	7.00	8.00	9.00

To determine the additional payoff, you go to the Google Widget that generates a random number between 0 and 9 presented below. I will ask you to report the random number that shows up the first time you use the Widget to generate it.

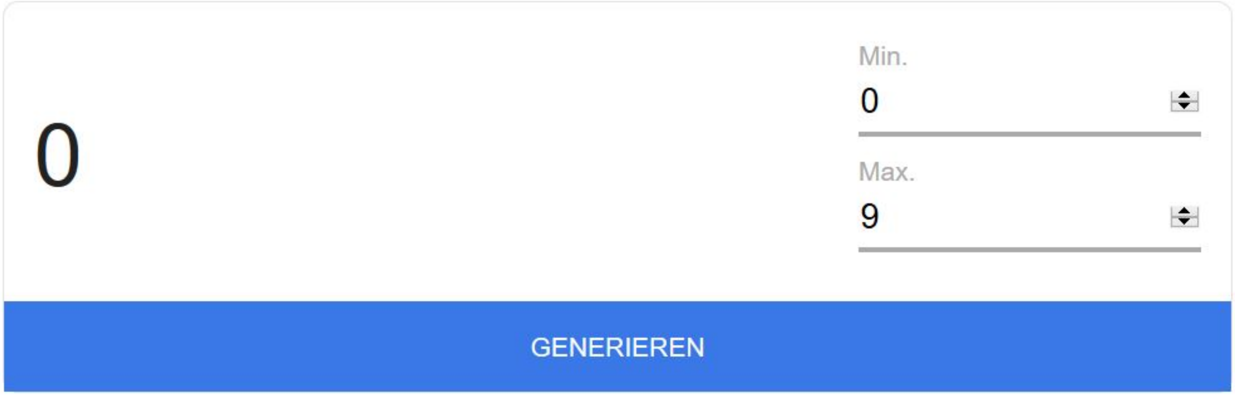

Figure A3: Random Number Generator

Note: Random Number Generator shown to participants.

In total you can earn between 6 CHF and 15 CHF in this experiment that takes about 25 minutes to finish. The structure of the experiment is as follows:

1. Personal survey
2. Questions regarding your decision on health insurance
3. Random number to determine additional payment
4. Two additional questionnaires

The information that I obtain will be used only for our research. It will be analyzed anonymously and will not be disclosed to third parties. The responses that you give cannot be connected to your identity by design of the experiment.

If at any time or for any reason you decide not to participate anymore, you are free to stop the experiment whether you have started or not.

Please make sure that you are in a quiet place without any distractions.

The payment for your participation will be sent to your bank account and I will ask you for your bank information on a separate website. Note that the money can only be transferred to a Swiss bank account, a PayPal or a Revolut account. Please have your IBAN or your PayPal e-mail address ready. Please note also that it is not possible for us to link your bank account information to the responses you give in this experiment as they are stored in a different database.

Please start this experiment only on your PC/mac and not on a mobile device.

Beliefs treatment

Welcome to our experiment! In this experiment I want you to answer a couple of survey questions as well as questions regarding your health insurance plan, for which you are being paid for. The questions are not meant to test you and there are no right or wrong answers. The experiment is being conducted by a research group at the University of St. Gallen. Your participation is completely voluntary. Please read the following explanations carefully.

For answering the short survey, I compensate you with a fixed participation fee of 6 CHF plus an additional payoff that is not the same for every participant. This additional payoff is determined by you guessing the behavior of participants in a similar, previously run experiment (from now on “reference experiment”). You will receive a more detailed description of the reference experiment at a later point in time.

In total you can earn between 6 CHF and 15 CHF in this experiment that takes about 25 minutes to finish. The structure of the experiment is as follows:

1. Personal survey
2. Questions regarding your decision on health insurance
3. Predict behavior of participants in previous experiment to determine additional payment
4. Two additional questionnaires

The information that I obtain will be used only for our research. It will be analyzed anonymously and will not be disclosed to third parties. The responses that you give cannot be connected to your identity by design of the experiment.

If at any time or for any reason you decide not to participate anymore, you are free to stop the experiment whether you have started or not.

Please make sure that you are in a quiet place without any distractions.

The payment for your participation will be sent to your bank account and I will ask you for your bank information on a separate website. Note that the money can only be transferred to a Swiss bank account, a PayPal or a Revolut account. Please have your IBAN or your PayPal e-mail address ready. Please note also that it is not possible for us to link your bank account information to the responses you give in this experiment as they are stored in a different database.

Please start this experiment only on your PC/mac and not on a mobile device.

6.3 Decision task

Decision task random number treatment

As you remember from the instructions, the payment for participating in this experiment is not the same for every participant. Chance determines your own additional payoff by you looking up a random number.

Please open another webpage and go to www.google.ch or directly visit LINK. Then search for “random number between 0 and 9”. Use the Google Widget that appears first in the search list to generate the number.

If you have consulted the page, please press NEXT.

Random number:	0	1	2	3	4	5	6	7	8	9
Resulting additional payoff (CHF):	0.00	1.00	2.00	3.00	4.00	5.00	6.00	7.00	8.00	9.00

Additional payment random number treatment

Please report the number that was generated by the random number generator.

Random number:	0	1	2	3	4	5	6	7	8	9
Resulting additional payoff (CHF):	0.00	1.00	2.00	3.00	4.00	5.00	6.00	7.00	8.00	9.00

Decision task financial market treatment

As you remember from the instructions, the payment for participating in this experiment is not the same for every participant. Chance determines your own additional payoff by you looking up the current value of the second decimal place of the stock index of your choice.

Please open another webpage and go to www.google.ch or directly visit LINK. Then search for either SMI if you want to use the Swiss Market Index or DAX if you want to use the DAX Performance Index. Use the Google Widget that appears first in the search list to lookup the second decimal place of the selected index value.

If you have consulted the page, please press NEXT.

Current value of second decimal digit of stock index:	0	1	2	3	4	5	6	7	8	9
Resulting additional payoff (CHF):	0.00	1.00	2.00	3.00	4.00	5.00	6.00	7.00	8.00	9.00

Additional payment financial market treatment

Please report the value that showed up in the second decimal place of the selected index.

Current value of second decimal digit of stock index:	0	1	2	3	4	5	6	7	8	9
Resulting additional payoff (CHF):	0.00	1.00	2.00	3.00	4.00	5.00	6.00	7.00	8.00	9.00

Decision task beliefs treatment

In the reference experiment, participant's payoff was determined randomly by the second decimal place of a stock market index. You can see how much additional payoff participants received conditional on the value of the second decimal of the index in the chart below.

Current value of second decimal digit of stock index:	0	1	2	3	4	5	6	7	8	9
Resulting additional payoff (CHF):	0.00	1.00	2.00	3.00	4.00	5.00	6.00	7.00	8.00	9.00

Participants were instructed to open another webpage and visit either LINK to SMI or LINK to DAX. Participants were instructed to use the Google Widget that appears first on the search list to lookup the second decimal place of the selected index value. I did not know about their choice of the index. I asked participants to report the current value of the second decimal place of the value of the selected index (marked here with a red circle). This number changes frequently and I asked participants to report only the value in the particular moment when they first looked at it once I had asked them to do so.

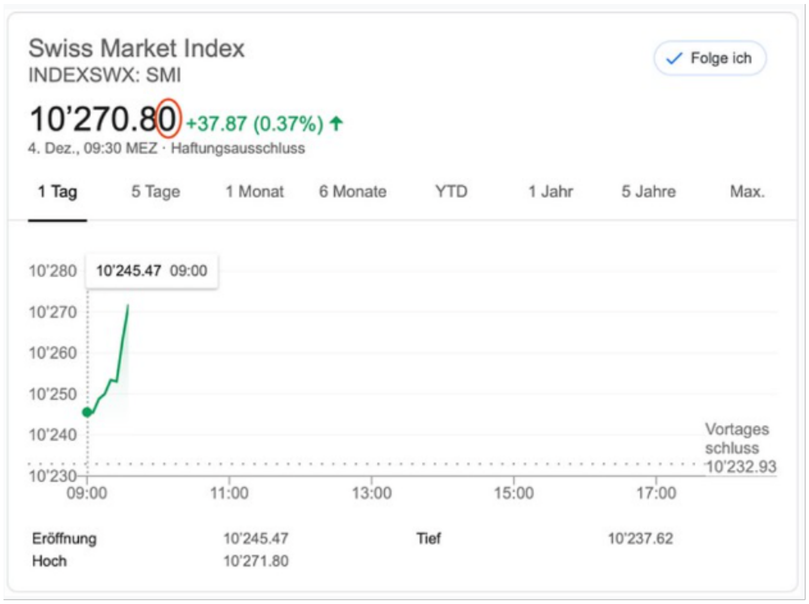

Figure A4: Google Widget for SMI

Note: Google Widget for SMI shown to participants.

For example, if the SMI value was 10’270.80, as in the example above, the correctly reported value was 0 and thus provided an additional payoff of 0 CHF. Note that nobody could detect whether their reported value was true or not.

Decision task beliefs treatment

I now ask you to assess behavior in the previously described reference experiment. With your assessment you can earn additional money. Please assess the payment (in CHF) of the participants of the reference experiment. If your assessment is correct you will receive CHF 9. For every percentage point you differ from the correct fraction, your payoff will be reduced by 10 Rappen. The smallest possible payment is CHF 1.

What percentage of participants received a payment of...

CHF 0	________ %	CHF 5	________ %
CHF 1	________ %	CHF 6	________ %
CHF 2	________ %	CHF 7	________ %
CHF 3	________ %	CHF 8	________ %
CHF 4	________ %	CHF 9	________ %

How sure are you about your assessment?

1 2 3 4 5

Figure A5: Beliefs

Note: I ask participants to guess behavior in a reference experiment.
